# Whole-Genome Sequencing of SARS-CoV-2 Infection in a Cluster of Immunocompromised Children in Indonesia

**DOI:** 10.3389/fmed.2022.835998

**Published:** 2022-03-04

**Authors:** Nina Dwi Putri, Edison Johar, Yora Permata Dewi, Nuri Dyah Indrasari, Dewi Wulandari, Merci Monica br Pasaribu, Teny Tjitra Sari, Fitri Prima Cakti, Madeline Ramdhani Jasin, Tartila Tartila, Frilasita Aisyah Yudhaputri, Safarina G. Malik, Khin Saw Aye Myint

**Affiliations:** ^1^Department of Paediatrics, Dr. Cipto Mangunkusumo National Central Hospital, Faculty of Medicine, Universitas Indonesia, Jakarta, Indonesia; ^2^Eijkman Institute for Molecular Biology, Jakarta, Indonesia; ^3^Department of Clinical Pathology, Dr. Cipto Mangunkusumo National Central Hospital, Faculty of Medicine, Universitas Indonesia, Jakarta, Indonesia

**Keywords:** COVID-19, SARS-CoV-2, quasispecies, whole-genome sequencing, hospital-acquired infection, children, immunocompromised, Indonesia

## Abstract

**Background:**

Thus far, Indonesia has recorded over 4,000,000 confirmed COVID-19 cases and 144,000 fatalities; 12.8% of cases have been in children under 18 years. Whole-genome viral sequencing (WGS) of severe acute respiratory syndrome coronavirus 2 (SARS-CoV-2) has been demonstrated to help differentiate hospital-acquired infection from community-acquired coronavirus disease 2019 (COVID-19) infection. Our study highlighted the use of WGS to investigate the origin of infection among pediatric oncology patients in Jakarta. The aim of our study was to evaluate clinical and laboratory characteristics and also the efficacy of using WGS to confirm hospital-acquired COVID-19 infection in a cluster of immunocompromised children within a single ward of a tertiary hospital in metropolitan Jakarta based on quasispecies, viral load, and admission dates.

**Method:**

Real-time reverse-transcription polymerase chain reaction (RT-PCR) from nasopharyngeal (NP) swabs was used to diagnose the patients and also guardians and healthcare workers (HCWs) in the ward, followed by WGS of RT-PCR positive cases to establish their phylogenetic relationships.

**Result:**

Using WGS, we showed that SARS-CoV-2 transmission in a cluster of children with underlying malignancy was characterized by high similarity of whole virus genome, which suggests nosocomial transmission.

## Introduction

Severe acute respiratory syndrome coronavirus 2 (SARS-CoV-2) is a highly contagious virus with a potential for outbreaks in healthcare institutions (1). Hospital-acquired infections of SARS-CoV-2 are rarely reported in children (2), most of whom were infected from household contacts (3). Although COVID-19 has often been reported as asymptomatic or manifested as mild-to-moderate upper respiratory tract infections (4), children with significant comorbidities are more prone to severe infection and poor prognosis (5).

Although whole-genome sequencing (WGS) is an effective tool to incriminate hospital-acquired infections (6), there have been relatively few reports of it being used in hospital SARS-CoV-2 outbreaks, especially in developing countries, such as Indonesia, where comprehensive and systematic contact tracing is challenging.

Notably, RNA viruses, which have both high replication rates and limited genetic proofreading, mutate to suit the new environment of new hosts (7, 8). Consequently, a pool of highly related mutants within the host, termed quasispecies, is created (7). This phenomenon has been documented in SARS-CoV-2 infections (9–13), with quasispecies diversifying as the virus resides longer in the host (12, 13). Transmission of the virus from host to host brings not only dominant mutants, but also a pool of minor mutants. In the next host, the transmitted pool of mutants experiences a transmission bottleneck, with only a handful of mutants fitting the new host, as observed previously in household SARS-CoV-2 transmission (12), and in influenza A virus (14, 15).

Consensus sequences portray master or dominant sequences, which provide a picture of genetic relatedness within an infection cluster, but are unable to capture the dynamics of virus population or sequence of infection. On the other hand, quasispecies dynamics could be depicted by highlighting active mutation sites even though it is difficult to separate the pool of highly related mutants into individual virus entity. In our study, we highlighted the use of quasispecies approach which could provide more insight into the potential source and sequence of infection in a hospital outbreak of SARS-CoV-2 in a cluster of children considered to be immunocompromised due to treatment for malignancies.

## Methods

This study was conducted in a general pediatric ward at Dr. Cipto Mangunkusomo Hospital, a tertiary care hospital in DKI Jakarta, Indonesia, from end December 2020 to March 2021. The study protocol was approved by the Eijkman Institute Research Ethics Committee (Approval Number 127) and FKUI-RSCM Health Research Ethics Committee (Ethical approval number KET-596/UN2.F1/ETIK/PPM.00.02/2020).

Nasopharyngeal (NP) swab specimens were collected on February 2 and 3, 2021 from the cluster of COVID-19 patients with malignancies, patients' guardians, and healthcare workers (HCWs). Samples were tested for RT-PCR in Clinical Pathology Laboratory of Integrated Laboratory Dr. Cipto Mangunkusumo Hospital. The second specimen was requested for WGS characterization on February 4, 2021. Subsequent swabs were not obtained to determine the duration of viral shedding due to the critical nature of the disease in the affected children. Data on clinical manifestations were obtained, together with routine hematology, homeostasis function, inflammatory markers, and chest X-ray (CXR) investigations from all positive cases.

Nucleic acid amplification test was performed on the specimens prior to sequencing (16). WGS was performed using ARTIC Network multiplex PCR method with version 3 primers (17). Library was prepared with Illumina TruSeq Nano DNA Library Prep kit and sequenced using Illumina MiSeq with 600 cycles kit. 30 bp were trimmed from 3′ and 5′ ends of each raw read using Geneious v2021.1.1 (https://www.geneious.com). The resulting reads were filtered using BBDuk v38.84 (18); only sequencing reads with length of ≥50 bp and Phred quality score of ≥30 were included in genome assembly using bowtie2 v2.3.0 (19). Consensus sequences were determined by representative bases with frequency ≥50%. Resulting sequences were deposited in GISAID (https://www.gisaid.org) with accession numbers of EPI_ISL_8540880, EPI_ISL_8540881, EPI_ISL_8540882, EPI_ISL_8540883, and EPI_ISL_8542984. These were aligned with sequences of PANGO lineage B.1.470 with high genome coverage available in GISAID (https://www.gisaid.org) and SARS-CoV-2 reference genome (Wuhan Hu-1, NC_045512.2, NCBI, https://www.ncbi.nlm.nih.gov/genome) using MAFFT v7.450 (20). A phylogenetic tree was generated using FastTree v2.1.12 GTR model with pseudocount (21).

To identify active mutation sites, 30 bp were trimmed from 3′ and 5′ ends of each raw read using Geneious v2021.1.1. The resulting reads were filtered using BBDuk v38.84; only sequencing reads with length of ≥50 bp and Phred quality score of ≥30 were mapped to SARS-CoV-2 reference genome using bowtie2 v2.3.0. Consensus sequences were generated by increasing representative base threshold to ≥95%. Any nucleotides with IUPAC ambiguity codes were defined as active mutation sites, and base composition was collected from the corresponding nucleotides using contig view of Geneious v2021.1.1. Only those with depth ≥100 reads were included in further active mutation site analysis. All bioinformatic tools used in this study were run in Geneious v2021.1.1.

## Results

Five immunocompromised children admitted for hematology–oncology disorders sharing a 6-patient room in a general pediatric ward at the Dr. Cipto Mangunkusomo Hospital were diagnosed with COVID-19 infection in early February 2021. The 6 beds were only separated by curtains for privacy. They were primarily admitted for the relapse of their underlying conditions (four with acute myeloid leukemia and one with Ewing's sarcoma); during that period, patients were screened for COVID-19 based on the symptoms in the emergency department and rapid SARS-CoV-2 antigen test before admitted to the ward. Only patient with the clinical symptoms of COVID-19 were tested with SARS-CoV-2 RT-PCR and admitted to the isolation ward. Mask wearing was mandatory to guardians but not for children, and the guardians were also allowed to move freely in and out of hospital. There was no history of travel nor known contact with a positive case for any of the children for the 2 weeks prior onset of illness.

The first child (case 3) admitted with fever and abdominal pain. During follow-up, she developed COVID-19-related symptoms that include fever and cough and was confirmed by RT-PCR on day 8. Additional cases were detected following contact tracing with RT-PCR including the remaining four children and one guardian of case 5. HCWs and guardians attending the children were asymptomatic. All staff members were negative for SARS-CoV-2.

Demographics, clinical characteristics, admission and SARS-CoV-2 PCR confirmation, comorbidities, laboratory investigations including chest imaging, treatment, and outcome are detailed in Table 1 and Supplementary Table S1. Cases 1 and 4 were discharged and readmitted after 1 and 2 weeks, respectively. Peripheral blood evaluation revealed that leucopenia, the most common white cell abnormality associated with children with COVID-19 (22), was seen in three patients (cases 2, 4, and 5); lymphopenia (23) and neutrophilia (24, 25) possible markers of severity were seen in two patients each [(cases 3, 5) and (cases 1, 3), respectively]. High total white cell (leucocytosis), lymphocyte counts (lymphocytosis), and thrombocytopenia associated with hematological malignancies (26) were seen only in a single patient (case 3), two patients (cases 2, 4), and three patients (cases 2, 4, and 5), respectively. Hypercoagulability (as evidenced by raised levels of D-dimer) reported in severe COVID-19 and multisystem inflammatory syndrome in children (MIS-C) (27) was seen in all four patients with available data (cases 1, 2, 3, and 5). C-reactive protein (CRP), an inflammatory marker, documented to be significantly increased in hematological malignancies (28) was seen in three patients (cases 1, 2, and 3). CXR confirmed pneumonia in three patients (cases 2, 3, and 4); consolidation, ground glass opacities, interstitial infiltrate, and pleural effusion being the common CXR findings. Blood, urine, and sputum culture were performed per standard of care. One child (case 5) with sepsis and systemic fungal infection was also diagnosed with MIS-C with raised CRP, procalcitonin, troponin, D-dimers, and fibrinogen. All five children had critical SARS-CoV-2 infection, and four succumbed to the illness.

**Table 1:** Symptoms, clinical, radiological features, treatments, and outcomes of the pediatric patients.

**Parameter**	**Case 1**	**Case 2**	**Case 3**	**Case 4**	**Case 5**
**Demographic**					
Age (years)	14	1	7	2	10
Gender	Female	Male	Female	Male	Male
Comorbidity	AML, septic shock, systemic fungal infection	AML, hypovolemic shock, gastroenteritis	Ewing's sarcoma, superior vena cava syndrome, sepsis	AML, sepsis	AML, sepsis, MISC, systemic fungal infection
**Presenting symptoms, clinical, and radiological features**					
Fever	+	+	+	+	+
Cough	+	–	–	–	–
Diarrhea	–	–	–	+	–
Nausea	+	–	–	–	–
Vomiting	+	–	–	–	–
Myalgia	–	+	+	+	+
Hematochezia	–	+	–	–	–
Hematemesis	–	+	–	–	–
Melena	–	+	–	–	–
Abdominal pain	–	–	+	–	–
Anorexia	–	–	+	–	–
Chest X-ray	No abnormality detected	Pneumonia	Right pleura effusion, pneumonia	Pneumonia	No abnormality detected
Treatment	Chemotherapy (cytarabine, carboplatin, etoposide, methotrexate), dexamethasone, ceftazidime, IV meropenem, IV imipenem-cilastatin, favipiravir, IV remdesivir	TC and PRC transfusion	Dexamethasone, methylprednisolone, amlodipine, furosemide, lovenox, meropenem, metronidazole, cefepime, ceftriaxone, remdesivir	Chemotherapy, dexamethasone, TC transfusion, amikacin, metronidazole, fluconazole, ceftazidime, meropenem, tygacil, cefotaxime, cefepime, remdesivir	Chemotherapy, morphine, sertraline, TC and PRC transfusion, mycamine, amphotericin B, methylprednisolone, dexamethasone, lovenox, meropenem, levofloxacin, ceftazidime, favipiravir, acyclovir, IVIG
Outcome	Discharged	Fatal	Fatal	Fatal	Fatal

*AML, acute myeloid leukemia; IV, intravenous; IVIG, intravenous immunoglobulin; MISC, multisystem inflammatory syndrome; PRC, packed red cells; TC, thrombocyte concentrates*.

### Molecular Characterization

**Figure 1:**
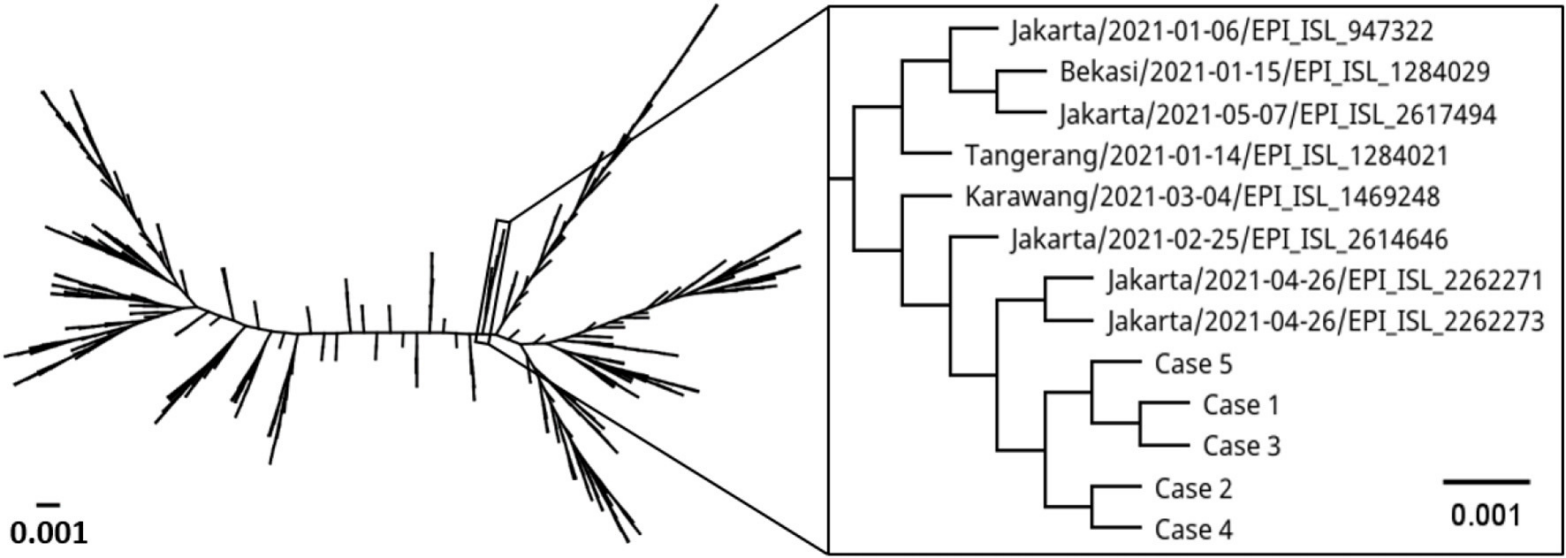
Phylogenetic tree comparing whole genome of SARS-CoV-2 from the five pediatric cases with SARS-CoV-2 lineage B.1.470 from around the world and reference genome (Wuhan Hu1). Label indicates collection site/collection date/GISAID accession number. The scale bar represents the number of nucleotide substitution per site.

Samples from the five pediatric patients were subjected to SARS-CoV-2 WGS. A number of SARS-CoV-2-specific reads with Phred quality score of ≥30 in the 5 cases were 2,341,675 reads (mean coverage: 12,465 reads; range 6–47,663 reads), 1,163,149 (mean: 4,095; 6–21,364), 1,024,727 (mean: 3,436; 8–14,440), 2,168,464 (mean: 12,693; 0–112,052), and 2,543,718 (mean: 13,771; 22–57,918), respectively. Whole-genome sequences were successfully recovered from the specimens with genome coverage of 99.6, 99.1, 99.7, 90.0, and 99.6%, respectively. The sequences belonging to PANGO lineage B.1.470 were then aligned with 603 sequences of B.1.470 and SARS-CoV-2 reference genome (Wuhan-Hu1). The sequences formed a genetic clade with cases from Jakarta and immediate surrounding areas (in box, Figure 1). The first subcluster consisted of cases 1, 3, and 5; cases 2 and 4 formed another subcluster.

We observed a few mutations in the genomes compared to Wuhan-Hu1 (Table 2). A total of 39 mutations were identified with 30 mutations found in all cases, and 9 mutations only found in some cases. The majority (53.9%) of the changes were a substitution to thymine base. Case 1 had one non-synonymous mutation each in envelope and spike, and one synonymous mutation in nsp4. Case 2 had one non-synonymous mutation in nsp2, and another in nsp4. Case 3 had one synonymous mutation each in nsp4 and spike. Case 4 was presented with one non-synonymous mutation each in nsp2 and spike, two synonymous mutations in nsp4, and a thymine base insertion in nsp3 gene. There was only one synonymous mutation in nsp4 observed in case 5. Moreover, there were two mutations shared among the cases; one synonymous mutation in nsp4 gene shared among cases 1, 3, 4, and 5 and a non-synonymous mutation in nsp2 gene of cases 2 and 4. In addition, we observed a rare C9565T substitution in nsp4 gene in four of the five cases. The mutation was reported in only 5.5% (33/603) of the analyzed B.1.470 sequences. None of the cases in the immediate genetic clade had the mutation (in box, Figure 1). High degree of similarity in mutations between cases is suggestive of hospital-acquired transmission.

**Table 2:** List of mutations based on consensus sequences of the five pediatric cases.

**Genome Position**	**Gene**	**Ref.^a^**	**Case**					**Type of mutation^b^**	**Amino acid change**
			**1**	**2**	**3**	**4**	**5**		
241	5' UTR	C	T	T	T	T	T	S	
1454	nsp2	C	G	G	G	G	G	NS	L → V
1613		C	C	**A**	C	**A**	C	NS	L → I
3004	nsp3	G	T	T	T	T	T	NS	E → D
3037		C	T	T	T	T	T	S	
4921		N	N	N	N	**T**	N	InDel	L → F, premature stop
6778		T	C	C	C	C	C	S	
9209	nsp4	G	G	**C**	G	G	G	S	
9483		A	A	A	A	**T**	A	S	
9565		C	**T**	C	**T**	**T**	**T**	S	
11288	nsp6	T	N	N	N	N	N	InDel	
11289		C	N	N	N	N	N	InDel	
11290		T	N	N	N	N	N	InDel	
11291		G	N	N	N	N	N	InDel	
11292		G	N	N	N	N	N	InDel	
11293		T	N	N	N	N	N	InDel	
11294		T	N	N	N	N	N	InDel	
11295		T	N	N	N	N	N	InDel	
11296		T	N	N	N	N	N	InDel	
14120	RdRP	C	T	T	T	T	T	NS	P → L
14408		C	T	T	T	T	T	NS	P → L
17421	nsp13	C	T	T	T	T	T	S	
18315	nsp14	G	A	A	A	A	A	S	
18877		C	T	T	T	T	T	S	
21597	S	C	T	T	T	T	T	NS	S → F
21794		A	A	A	A	**G**	A	NS	R → G
22323		C	**T**	C	C	*Gap*	C	NS	S → F
23403		A	G	G	G	G	G	NS	D → G
23929		C	C	C	**T**	C	C	S	
25563	ORF3a	G	T	T	T	T	T	NS	Q → H
25855		G	C	C	C	C	C	NS	D → H
26051		G	T	T	T	T	T	NS	S → I
26456	E	C	**T**	C	C	C	C	NS	P → L
26681	M	C	T	T	T	T	T	NS	P → S
26735		C	T	T	T	T	T	NS	Q → Stop
27870	ORF7b	G	T	T	T	T	T	NS	Stop → E
28887	N	C	T	T	T	T	T	NS	T → I
29311		C	T	T	T	T	T	S	
29754	3'UTR	C	T	*Gap*	T	*Gap*	T	S	

a *Ref.: Reference genome (Wuhan Hu1).*

b *NS, non-synonymous; S, synonymous; InDel, insertion/deletion. The bold values indicated to highlight value differences*.

### Possible Source and Sequence of Infection

We attempted to reconstruct the sequence of infection based on active mutation sites, patient admission time, and viral load. To identify the sites, representative base threshold was increased to ≥95%, and bases with depth <100 reads were excluded from the analysis to avoid data distortion. Active mutation identified in the 5 cases were 7, 21, 14, 13, and 6 sites, respectively (Supplementary Table S2). The majority of sites (>70%) resided within genes encoding non-structural proteins. There were six shared active sites at genome position of 1613, 9565, 11286, 11287, 11511, and 28254, all in genes for non-structural proteins (Table 3).

**Table 3:** List of shared active mutation sites of the five pediatric cases with base composition in percentage of total reads.

**Gene**	**Position**	**Base**	**Case**				
			**1**	**2**	**3**	**4**	**5**
nsp2	1613	A	0	70.7	0.1	97.4	0
		T	0	0	0.1	0	0
		G	0	0	0.1	0	0
		C	100	29.3	99.8	2.6	100
		Depth	10,346	1,078	1,992	114	10,139
nsp4	9565	A	0	0	0	0	0
		T	83.5	48.0	94.1	99.9	79.9
		G	0	0	0	0	0
		C	16.5	52.0	5.9	0.1	20.1
		Depth	13,893	13,253	6,656	18,554	15,709
nsp6	11286	A	1.7	3.1	3.3	1.4	2.0
		T	94.9	90.5	90.9	96.3	94.0
		G	3.4	6.4	5.7	2.3	4.0
		C	0	0	0	0	0
		Depth	20,745	12,194	9,999	36,273	20,443
nsp6	11287	A	4.3	8	7.7	2.8	5.1
		T	2.1	3.5	3.7	1.8	2.3
		G	93.6	88.6	88.6	95.4	92.6
		C	0	0	0	0	0
		Depth	20,788	13,500	10,009	36,375	20,462
nsp6	11511	A	0	0	0	0	0
		T	0	20.4	0	27.1	0
		G	0	0	0	0	0
		C	100	79.6	100	72.9	99.9
		Depth	14,116	2,010	6,557	48,036	10,454
ORF8	28254	A	93.0	94.6	94.2	95.2	96.5
		T	0.1	0	0	0	0
		G	0.0	0	0	0	0
		C	3.3	1.4	1.4	3.7	1.5
		Del^a^	3.7	4.0	4.4	1.1	2.0
		Depth	17,168	4,656	5,297	82,051	20,152

a *Del, base deletion*.

Case 2 had the most active mutation sites and thus presumed to be the source of the outbreak. This patient was admitted on January 18, 2021 for the underlying condition and likely was at that time infected with SARS-CoV-2. The virus might have infected case 5, who had been admitted on December 23. Case 1 was admitted into the room 2 days later (January 20, 2021) and could also have been infected by case 2. These speculations are supported by the similar viral load of case 1 (Ct value of 14.74) and case 5 (Ct value of 13.87), compared to a higher viral load (Ct value of 9.81) in case 2. Although case 4 was admitted on January 15, the patient appeared to be infected later, as indicated by much lower viral load at the time of sample collection (Ct value of 33.82). The C9565T mutation was observed in all cases, except case 2, in the consensus genome analysis. At the subconsensus sequence level, the substitution observed in case 2 had a proportion of 52.0% reads with cytosine and 48.0% with thymine. The other four cases exhibited low-base heterogeneity at the corresponding positions. We presume case 2 contributed the rare C9565T mutation. As the virus infected new hosts, that is, cases 1 and 5, a more favorable mutation could have prevailed. The mixture of high thymine (79.9–83.5%) and low cytosine (16.5–20.1%) in both cases demonstrated thymine gradually becoming a dominant base. In addition, thymine and cytosine in genome position 9565 were observed at similar proportion in cases 1 and 5, consistent with the conjecture of similar cases 1 and 5 infection time.

Case 3 was the last patient to be admitted on January 26, before the reported outbreak in the room. Assuming the average virus incubation period of 6 days (29), cases 1, 2, and 5 could be in the infectious stage at the time of case 3 admission. By taking into account the mutation spectra of ORF7b (position 28254), case 3 had base heterogeneity more similar to cases 1 and 2. We also observed the base heterogeneity in nsp6 (position 11286-7) in the cases. Case 3 had more similar profile to case 2 than case 1. Thus, case 3 was likely infected by virus from case 2.

Case 4 was likely infected later than case 3. Viral load in case 4 (Ct value of 33.82) was at least 10 times lower than case 3 (Ct value of 29.58), which indicates later infection. Cases 2 and 4 shared two unique mutation spectrum at genome position 1613 (nsp2) and 11511 (nsp6). It suggested that case 4 was likely infected by case 2, presumably gaining the mutations after cases 1, 3, and 5 infections. Furthermore, both cases 3 and 4 had low viral load at the time of sampling, which indicates that the cases were infected later than cases 1 and 5.

We observed similar level of base heterogeneity across all cases in genome position 11286 (nsp6), 11287 (nsp6), and 28254 (ORF8). A small subset (1.1–4.4%) of total reads in the ORF8 had one base deletion at the end of the gene, which results in amino acid change from isoleucine to serine. In addition, we noticed that case 4 had three sites with thymine insertion: genome position 4921 (nsp3, 62.2% reads with insertion), 11179 (nsp6, 41.0%), and 27821 (ORF7b, 11.9%). The insertions caused frameshift mutations, which results in truncated proteins.

## Discussion

Our report described a linked cluster of B.1.470 infections, clinical manifestations, and outcome in a cohort of immunocompromised pediatric patients with oncological comorbidity in Jakarta during the second year of COVID-19 pandemic. The first case was diagnosed with hospital-associated SARS-CoV-2 eight days postadmission for her underlying condition. The commonest symptom of COVID-19 reported in pediatric cancer patients was fever followed by cough (30) the same as manifested in case 3. However, most of our study patients had masked symptoms of COVID-19, which was to be expected with the oncology comorbidity and therapy. Although children with cancer are vulnerable to COVID-19 infection due to immunosuppression associated with the disease and its treatment (24), the impact of SARS-CoV-2 on pediatric patients with hematological malignancies and solid tumors in low- and middle-income countries has been rarely reported. Pediatric cancer patients with SARS-CoV-2 infection were associated with milder infection than adults (31); however, they were reported to have a more severe disease and mortality compared to the general population (30). In our study, 4 of 5 in our cluster had succumbed to the illness likely due to complications of the underlying condition or opportunistic infection rather than the SARS-CoV-2 infection.

To prevent outbreaks of COVID-19 in a healthcare setting, it is important to investigate patients, HCWs, and close contacts with PCR test including asymptomatic individuals. Our study had undetermined source of infection as guardians and visitors were not vigorously screened during that period. Although the guardian of one immunocompromised child was positive by the onsite RT-PCR, the specimen was not saved for further genome sequencing. The majority of healthcare-associated SARS-CoV-2 infections were due to patient-to-patient or HCW-to-patient transmission (1). SARS-CoV-2 hospital outbreaks may often originate from HCWs (32); those attending the cluster were tested negative once and not serially checked, which did not provide enough evidence to exclude SARS-CoV-2 transmission from HCWs. With the strain from one positive guardian unavailable for WGS, genomic links between patients and other sources were also not thoroughly explored. Moreover, three confirmed patients had Ct ≤ 25, which suggests high viral loads, with a potential for patient-to-patient spread as studies have shown links between high viral loads and an increased transmission risk (33).

Symptoms of SARS-CoV-2 infection can overlap with exacerbation of the primary disorder, which suggests that routine screening is crucial in this vulnerable population for any viral respiratory outbreaks. In addition, testing of stool specimen in combination with chest CT is recommended to confirm COVID-19 infection in those with negative swabs as the infection can be masked by malignancy (34, 35). It has been suggested that immunodeficient individuals may have prolonged viral shedding and potentially be contagious for longer duration (36); however, in our study, subsequent respiratory specimens were not obtained due to the critical nature of the illness.

We successfully recovered SARS-CoV-2 genomes from the patients, assigned as PANGO lineage B.1.470. The variant, first identified a year ago in Indonesia, is now in circulation globally. More than 800 complete WGS of B.1.470 have been submitted to GISAID, mostly from Indonesia (>70%), including those from travelers visiting the country. Although all five immunocompromised children acquired the infection with high mortality (80%); transmissibility, severity, and neutralizing antibody response of this strain is not well studied. One study of 41 patients with B.1.470 infection showed 19.5% as asymptomatic, 31.7% as mild severity, and 48.8% as moderate severity (37). None were presented with severe clinical manifestations, but the patients were relatively young with median age of 31 years (range: 27.5–41.0). From our sequencing data, we identified 39 mutations compared to SARS-CoV-2 reference genome. Nine of the mutations were unique to the genetic clade of the five patients, which consisted of four synonymous mutations, four non-synonymous mutations, and one thymine insertion. A non-synonymous mutation of L270I in nsp2 has not been reported, and R78G in spike has been reported in GISAID, but without functional study. Another mutation was S254F in the N-terminal of spike for which no structural and antigenic changes were reported (38). Additional mutation was observed in envelope gene, which results in amino acid change from proline to leucine at position 71. The mutation has been reported to occur at a low frequency, but it appeared to produce no functional changes to the protein (39). Finally, thymine base insertion in nsp3 was observed in case 4, which results in premature protein translation. The truncated protein lost papain-like protease motif that has been described to involve in modulating host antiviral response (40). However, overall impact of the nine mutations remained unknown in regard to disease severity and virus fitness. Interestingly, we also observed that the majority of the mutations were substitution mutations, with a majority from cytosine to thymine base, similar to the analysis from the early stage of pandemic (41). It was hypothesized the changes fit mutational pattern mediated by the host APOBEC family proteins which are known to possess mRNA-editing activities (41, 42).

We also explored the use of quasispecies to determine possible source of infection. Specimens in this study were collected from the patients at the same day and in the early symptom onset. It has been documented in a study of one patient that intrahost SARS-CoV-2 virus quasispecies composition changed day by day (11). We also observed transmission bottleneck as certain variants became more dominant after jumping to other patients, as exemplified by C9565T variant in cases 1 and 5 following infection from case 2. Taken together with quasispecies dynamics, it allowed us to speculate on possible order of infection. We observed with interest that cases 1 and 5 did not develop pneumonia and had low quasispecies variants at 7 and 6, respectively. On the other hand, cases 2, 3, and 4 did develop pneumonia and had high quasispecies variants at 21, 14, and 13, respectively. Although it is difficult to rule out pneumonia caused by opportunistic pathogens due to the immunocompromised nature of the study patients, a link between disease severity and number of quasispecies variants has been established in SARS-CoV-2 (13, 43), and other RNA viruses (44). In addition, immunocompromised patients are at risk of prolonged infection, which allows the virus to develop detrimental mutations, which could lead to antibody evasion and potentially increased disease burden (45, 46).

Our study had a few limitations: 1. The study assumed a single introduction of one strain of virus into the shared ward; the possibility of multiple strains introduction could not be excluded as guardians' movements were not restricted prior to the outbreak. However, this was unlikely, as all five sequences from the patients formed one genetic clade, and the infection time presumably was not long enough to allow genome recombination from multiple virus strains. 2. Viral load comparison between samples may not represent true viral load due to challenges obtaining NP samples from young children. 3. Our study lacked systematic and comprehensive specimen collection that could strengthen the epidemiological link between patients. In addition, the cluster of immunocompromised children with malignancy was limited to five; the study should be expanded to include more patients to draw meaningful conclusions. 4. We could only investigate specimens at one-time point and did not have access to the subsequent specimens to study the dynamics of viral quasispecies. Gradual change of quasispecies variants in hosts infected by the same virus strain has been noted in HIV-1 infection, with high relatedness in early infection and progressively becoming less related (47). 5. Some quasispecies variants might be underrepresented or not visible in this study, as we did not perform ultra-deep sequencing.

## Conclusion

In this study, WGS of a linked cluster of SARS-CoV-2 in immunocompromised children in a single ward demonstrated distinctive viral genomic mutations in the hospital cluster that indicated hospital-acquired transmission in a shared ward. Aggressive and routine contact tracing, and also widespread testing of patients and HCWs, including asymptomatic individuals, is essential to limit hospital-associated transmission of COVID-19 including the new variants. Our study also highlighted the use of viral quasi-species to establish epidemiological link between patients in a shared ward.

## Data Availability Statement

The datasets presented in this study can be found in online repositories. The names of the repository/repositories and accession number(s) can be found below: GISAID with accession numbers for the five cases: EPI_ISL_8540880, EPI_ISL_8540881, EPI_ISL_8540882, EPI_ISL_8540883, and EPI_ISL_8542984.

## Ethics Statement

The studies involving human participants were reviewed and approved by Eijkman Institute Research Ethics Committee (Approval Number: 127) and FKUI-RSCM Health Research Ethics Committee (Ethical Approval Number: KET-596/UN2.F1/ETIK/PPM.00.02/2020). Written informed consent to participate in this study was provided by the participants' legal guardian/next of kin.

## Author Contributions

NP, NI, DW, MP, TS, FC, MJ, and TT collected the clinical data. EJ and NP performed data analysis and interpretation. EJ, KM, and YD wrote the first draft. NP, EJ, YD, NI, DW, MP, TS, FC, MJ, TT, FY, SM, and KM revised the final manuscript. All authors read and approved the final manuscript.

## Funding

This work was supported by the Ministry of Research and Technology/National Research and Innovation Agency, Republic of Indonesia and U.S. Centers for Disease Control and Prevention (US CDC).

## Conflict of Interest

The authors declare that the research was conducted in the absence of any commercial or financial relationships that could be construed as a potential conflict of interest.

## Publisher's Note

All claims expressed in this article are solely those of the authors and do not necessarily represent those of their affiliated organizations, or those of the publisher, the editors and the reviewers. Any product that may be evaluated in this article, or claim that may be made by its manufacturer, is not guaranteed or endorsed by the publisher.
