# Supplementary material for: Whole-Genome Sequencing of SARS-CoV-2 Infection in a Cluster of Immunocompromised Children in Indonesia
Source: Front Med (Lausanne). 2022 Mar 4;9:835998. doi: 10.3389/fmed.2022.835998 (PMC8930830; doi:10.3389/fmed.2022.835998)
Supplement: Supplementary file 1 [file Table_1.docx]

Supplementary Table 1. Laboratory findings of the pediatric patients

| **Parameter** | **Case 1** | **Case 2** | **Case 3** | **Case 4** | **Case 5** | **Ref value** |
| --- | --- | --- | --- | --- | --- | --- |
| **Laboratory investigations** | | | | | | |
| SARS-CoV-2 RT-PCR | Positive | Positive | Positive | Positive | Positive |  |
| Interval between admission and positive test (days) | 14 | 16 | 8 | 19 | 45 |  |
| **Blood test** | | | | | | |
| Hemoglobin (g/dL) | 9.6 | 8.9 | 9.4 | 7.7 | 12.3 | 11.5-15.0 |
| White blood cell (cells/µL) | 6,370 | 72 | 20,470 | 3,870 | 2,130 | 5,000-13,000 |
| Basophil (%) | 0.3 | 0 | 0.3 | 0 | 0 | 0-2 |
| Eosinophil (%) | 0 | 1.4 | 0 | 0 | 1 | 1-6 |
| Neutrophil (%) | 75.2 | 5.5 | 82.6 | 24 | 68 | 40-60 |
| Lymphocytes (%) | 24.3 | 90.3 | 7 | 61 | 18 | 20-40 |
| Monocytes (%) | 0.2 | 2.8 | 10.4 | 15 | 12 | 2-10 |
| Hematocrit (%) | 36.8 | 25.9 | 28.0 | 22.7 | 34.4 | 34-45 |
| Platelets (counts/µL) | 279,000 | 4,000 | 232,000 | 3,000 | 55,000 | 150,000-410,000 |
| Activated partial thromboplastin time (s) | ND | 42.8 | ND | ND | 36.2 | 31-47 |
| Prothrombin time (s) | ND | 15.4 | ND | ND | 12 | 11-12.5 |
| Fibrinogen (mg/dL) | ND | 631.9 | ND | ND | 396.2 | 200-400 |
| D-dimer (mg/L) | 1,830 | 5,900 | 12,710 | ND | 2,820 | <440 |
| Procalcitonin (ng/mL) | 2.21 | 0.39 | 28.59 | 0.46 | 0.29 | ≤0.05 |
| C-reactive protein (mg/L) | 140.1 | 318.7 | 338.7 | 35.2 | 18.9 | 0.0-3.0 |
| Troponin I (pg/mL) | ND | ND | 34.7 | ND | 45.8 | <34.2 |
